# The REthinking Clinical Trials Program Retreat 2023: Creating Partnerships to Optimize Quality Cancer Care

**DOI:** 10.3390/curroncol31030104

**Published:** 2024-03-06

**Authors:** Ana-Alicia Beltran-Bless, Mark Clemons, Lisa Vandermeer, Khaled El Emam, Terry L. Ng, Sharon McGee, Arif Ali Awan, Gregory Pond, Julie Renaud, Gwen Barton, Brian Hutton, Marie-France Savard

**Affiliations:** 1 Department of Medicine, Division of Medical Oncology, The Ottawa Hospital, University of Ottawa, Ottawa, ON K1H 8L6, Canada; abeltran@toh.ca (A.-A.B.-B.); mclemons@toh.ca (M.C.); teng@toh.ca (T.L.N.); shmcgee@toh.ca (S.M.); aawan@ohri.ca (A.A.A.); 2 Cancer Therapeutics Program, Ottawa Hospital Research Institute, Ottawa, ON K1H 8L6, Canada; lvandermeer@ohri.ca; 3 CHEO Research Institute, Ottawa, ON K1H 5B2, Canada; kelemam@ehealthinformation.ca; 4 Department of Oncology, McMaster University, Hamilton, ON L8S 4L8, Canada; gpond@mcmaster.ca; 5 Champlain Regional Cancer Program, Ottawa, ON K1H 8L6, Canada; jurenaud@toh.ca; 6 Psychosocial Oncology, Patient Engagement/Experience, Ottawa Hospital Cancer Centre, Ottawa, ON K1H 8L6, Canada; gwbarton@toh.ca; 7 Clinical Epidemiology Program, Ottawa Hospital Research Institute, Ottawa, ON K1H 8L6, Canada; bhutton@ohri.ca; 8 School of Epidemiology and Public Health, University of Ottawa, Ottawa, ON K1N 6N, Canada

**Keywords:** pragmatic clinical trials, quality of cancer care, patient experience

## Abstract

Patients, families, healthcare providers and funders face multiple comparable treatment options without knowing which provides the best quality of care. As a step towards improving this, the REthinking Clinical Trials (REaCT) pragmatic trials program started in 2014 to break down many of the traditional barriers to performing clinical trials. However, until other innovative methodologies become widely used, the impact of this program will remain limited. These innovations include the incorporation of near equivalence analyses and the incorporation of artificial intelligence (AI) into clinical trial design. Near equivalence analyses allow for the comparison of different treatments (drug and non-drug) using quality of life, toxicity, cost-effectiveness, and pharmacokinetic/pharmacodynamic data. AI offers unique opportunities to maximize the information gleaned from clinical trials, reduces sample size estimates, and can potentially “rescue” poorly accruing trials. On 2 May 2023, the first REaCT international symposium took place to connect clinicians and scientists, set goals and identify future avenues for investigator-led clinical trials. Here, we summarize the topics presented at this meeting to promote sharing and support other similarly motivated groups to learn and share their experiences.

## 1. Introduction

The development of new agents for cancer treatments is costly, with the average cost of developing a new drug estimated to be over a billion USD [1,2]. Thus, while many of these anticancer treatments are innovative and influential on medical practice, these costs indicate that cancer care is becoming increasingly unsustainable. This results in variability in oncology care becoming increasingly common between countries, centres and individual healthcare providers. Too often, patients, families, healthcare providers and funders face multiple treatment choices without knowing which one provides the best care. Unfortunately, clinical trials that answer pragmatic questions that can help standardize and optimize real-world clinical practice, such as “Which ones of the multiple standard treatments available optimize patient outcomes?” or “What is the optimal duration of treatment for patients?”, usually generate less interest and academic recognition than pharmaceutical-company-funded trials. As a result, conducting and funding such investigator-initiated trials have been challenging.

The REthinking Clinical Trials (REaCT) Program was created in 2014 to disrupt how cancer trials were conducted in Canada. It uses a rigorous methodology of surveys targeting patients and healthcare providers, systematic reviews and pragmatic clinical trials [3,4,5,6,7,8]. The REaCT Program is the largest pragmatic cancer clinical trial program in Canada. It has randomized patients to a broad range of clinical indications, reflecting not just different types of cancer but also different surgical, pathological and radiological approaches as well as adjuvant radiotherapy and systemic treatment, and supportive and palliative care options. The REaCT program follows a framework described previously [3]. A potential trial should be pragmatic, evaluate a setting in which there is clinical equipoise, use an integrative consent model, require no additional visits or tests over and above the standard of care, and make use of broad eligibility criteria. Additional data collection outside of standard practice should be limited. The trial must aim to improve patient care or resources and change clinical practice with a practical knowledge translation plan. All these features ensure that our trials are highly relevant to clinical practice and are performed efficiently, meaning that they are on time and on budget. As of December 2023, REaCT has conducted 27 trials at 20 Canadian centres with a total of over 4500 patients enrolled.

The 2023 REaCT Retreat was the inaugural Canadian meeting to review the past experiences and future avenues of the REaCT Program. The meeting was held in Ottawa on 2 May 2023, with featured speakers from across Canada. Topics of discussion ranged from a review of the REaCT Program and pragmatic trial design, knowledge translation, patient engagement, machine learning and artificial intelligence, the use of routinely collected administrative health data and dose optimization trials. The ultimate goal of the meeting was to see how different researchers and investigators could work together to improve the quality of cancer care globally.

## 2. Review of Recently Completed and Currently Accruing REaCT Studies

### 2.1. REaCT-Algorithm (PI: Arif Awan)

Physicians rely on both clinicopathologic factors, using publicly available risk tools such as PREDICT 2.1, and/or molecular tests, such as Oncotype DX^®^ (ODX), to decide whether there is a benefit in the administration of chemotherapy for patients with early-stage hormone receptor (HR)-positive, Her2-negative breast cancer. The best way to incorporate both tests still needs to be discovered, and it is unclear how the use of risk tools affects physicians’ decision making in ordering gene expression profiling tests.

The REaCT Algorithm study “Does use of PREDICT 2.1 Tool affect Oncotype DX^®^ recurrence score ordering? A multi-centre prospective cohort study” was presented [9]. This multi-center prospective trial assessed how clinicians use PREDICT 2.1 to guide their decision making when ordering ODX. The study’s team collected data on physicians’ use of PREDICT 2.1 and ODX tests for six months for all eligible patients. After six months, the team conducted an educational intervention to see if providing physicians with the PREDICT 2.1 results affected the frequency of their ODX requests. The primary outcome of this study was the proportion of patients for whom ODX was ordered, defined as the number of patients with ODX orders divided by the number of patients eligible for ODX testing. The eligibility criteria matched Ontario publicly funded testing indications at that time and included the following: patients who were ER positive, PR positive/negative, HER2 negative, lymph node negative or with micro-invasive disease, and those with a tumour >1 cm in size (or if ≤1 cm, it must be grade 2 or 3 or have lymph node micrometastasis). Patients were not eligible if they had received neoadjuvant chemotherapy or were diagnosed with recurrent breast cancer.

**Figure 1:**
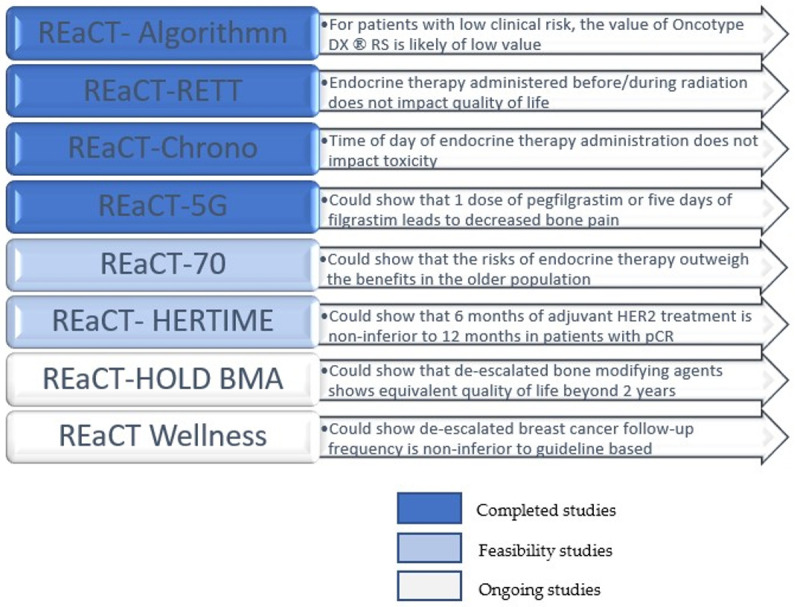
How REaCT studies can improve patient care.

Between 6 March 2020 and 19 November 2021, 602 patients across six cancer centres in Ontario, Canada were recruited. The results showed that the educational intervention did not impact requests for ODX (Table 1). For patients with low clinical risk, either by clinico-pathological features or by PREDICT 2.1, the probability of obtaining a high Oncotype DX recurrence score was substantially lower compared to that of patients with high-clinical-risk disease. The study suggests that the routine ordering of ODX for patients with low-clinical-risk disease is of low value for most patients without strong clinical evidence they would benefit from adjuvant chemotherapy, whereas patients with high-clinical-risk disease would be more likely to benefit from adjuvant chemotherapy and ODX can prevent them from being overtreated with chemotherapy [10] (Figure 1).

### 2.2. REaCT-RETT (PI: Sharon McGee)

The optimal timing of radiation therapy and starting endocrine therapy for early breast cancer (EBC) is unknown. Some concern exists regarding the concomitant administration of endocrine therapy throughout radiation due to the possible risk of increased toxicities. The REaCT-RETT study randomized patients to receive endocrine therapy concurrently with or sequentially after radiation [11,12]. The primary endpoint was endocrine therapy toxicity as per the change in the FACT-ES score. The hypothesis was that there was no difference in endocrine or radiation toxicity with the timing of administration of endocrine therapy.

The study recruited 262 patients across three sites. It found no difference in endocrine toxicity from the baseline to three months and no difference in quality of life, endocrine compliance or radiation toxicity at twelve months (Table 1, Figure 1). Therefore, the decision regarding the timing of endocrine therapy should be individualized for each patient with no increased concerns regarding toxicity [13].

### 2.3. REaCT-CHRONO (PI: Marie-France Savard)

The side effects of endocrine therapy are a common reason for non-adherence to adjuvant treatment [14]. No prospective evidence exists for whether the time of day of administration affects endocrine side effects. The REaCT-CHRONO study randomized patients receiving adjuvant endocrine therapy to either morning or evening doses of endocrine therapy [15]. The primary endpoint was endocrine therapy toxicity and tolerability measured by the change in the total Functional Assessment of Cancer Therapy-Endocrine Subscale (FACT-ES) score from the baseline to 12 weeks following the beginning of ET.

Between June 2021 and March 2022, 245 eligible patients were randomized (16). The results showed no significant difference in endocrine therapy toxicity and tolerability between the morning and evening timings of endocrine therapy (Table 1, Figure 1) (16). The secondary endpoint results, including the 12-month adherence rate and patient quality of life, were presented in a later symposium in 2023 [16].

### 2.4. REaCT-HER TIME (PI: Sharon McGee)

The decision regarding the optimal duration of adjuvant HER2 therapy remains controversial, and de-escalating HER2-targeted treatment has yet to become common practice [17,18]. The REaCT-HER TIME pilot study (NCT04928261) randomized patients with HER2+ early-stage breast cancer who had had upfront systemic treatment with no residual disease on surgical specimens to either six or twelve months of adjuvant HER2 treatment. The primary outcome was feasibility. The hypothesis is that patients with a pathological complete response (pCR) were sufficiently treated with six months of therapy. This study is currently open to accrual [19].

### 2.5. REaCT-5G (PI: Terry Ng)

Granulocyte colony-stimulating factor (G-CSF) is recommended for the primary prevention of febrile neutropenia (FN) in patients receiving chemotherapy. Because there is sufficient equipoise between the efficacy of five days of filgrastim (requires daily subcutaneous injections × 5–10 days after chemotherapy) and the efficacy of pegfilgrastim (a single injection after chemotherapy), we felt that if there were a significant difference in bone pain, a common side effect of G-CSF, then that would impact treatment decisions. We conducted REaCT-5G, a multi-centre, randomized controlled trial comparing bone pain experienced by patients receiving five days of filgrastim or a single dose of pegfilgrastim in patients with early breast cancer receiving neo-/adjuvant chemotherapy requiring primary febrile neutropenia prophylaxis. Participants were randomized to either one dose of pegfilgrastim (PEG) or five days of filgrastim (FIL) starting 24–48 h after each chemotherapy cycle [20]. The primary outcome was bone pain during cycle 1 of chemotherapy, 1 to 5 days after the G-CSF injection. The study accrued 233 patients at two sites between June 2021 and March 2023. The results showed no significant difference in patient-reported bone pain or patients’ quality of life between 5 days of FIL and a single dose of PEG and no difference in chemotherapy delay, dose reduction and premature discontinuation (Table 1, Figure 1). Interestingly, at the end of the study, patient preference for PEG increased in the PEG-treated group. In contrast, patient preference did not change significantly in the group that received five days of filgrastim [21].

### 2.6. REaCT-70 (PI: Marie-France Savard)

The optimal adjuvant strategy around the omission of radiotherapy and/or endocrine therapy in older patients who have undergone breast-conserving surgery is unclear. Most of the evidence has shown that the omission of RT does not affect outcomes, but there is a paucity of evidence regarding the omission of endocrine therapy [6]. The REaCT-70 pilot study (NCT04921137) randomizes patients over 70 years of age with lower-risk disease who have had optimal loco-regional therapy (i.e., a mastectomy or lumpectomy plus radiotherapy) to either receive or not receive endocrine therapy [22]. The hypothesis is that endocrine therapy’s risks and harms outweigh this population’s benefits (Figure 1). The primary endpoint was feasibility. This study was open at six sites and had accrued 72 out of 100 patients at the time of the REaCT meeting. The pilot subsequently completed its accrual in December 2023 and its results will be presented in 2024.

### 2.7. REaCT-HOLD BMA (PI: Terry Ng)

Bone-modifying agents (BMAs) are recommended in patients with metastatic breast cancer, metastatic castrate-resistant prostate cancer and bone metastases to reduce symptomatic skeletal events (SSEs), but randomized controlled trials have only evaluated the value of these agents during the first two years of treatment. Therefore, these agents’ optimal duration and frequency after the first two years have not been established. The REaCT-HOLD BMA trial is the first pragmatic multi-centre study to evaluate the optimal interval or frequency of BMA use after a minimum of two years of prior BMA treatment for bone metastases in patients with metastatic breast cancer and metastatic castrate-resistant prostate cancer. This non-inferiority study hypothesized that a less frequent administration of BMAs in the longer term will be non-inferior to administering BMAs more frequently (every 4 or 12 weeks) in terms of patients’ health-related quality of life (Figure 1). Study participants were randomly allocated to continue BMAs with the same administration schedule (every four or 12 weeks) or change the BMA frequency to every 24 weeks [23]. The study opened in 2020 and has enrolled 171 patients at five sites so far.

### 2.8. REaCT-Wellness (PI: Ana-Alicia Beltran-Bless)

The frequency and nature of breast cancer follow-up after patients complete the acute phase of their treatment vary between and within different institutions [24]. Guideline recommendations are not supported by any level I evidence. The REaCT Wellness study randomizes patients with early-stage breast cancer who have been referred to our survivorship program to either guideline-based survivorship care or an annual phone call post-mammogram with on-demand access if needed [25]. The primary outcome was patient quality of life as determined by the FACT-G questionnaire 1 year post-randomization. A total of 244 patients were accrued from September 2022 to March 2023. The study will be completed in March 2025.

## 3. Pragmatic Trial Designs: Understanding Key Statistical Concepts Presented by Gregory Pond

### 3.1. Per Protocol versus Intent-to-Treat Analyses

This session reviewed the main key statistical concepts important to the design of our pragmatic clinical trials. All REaCT trials are designed using an intention-to-treat (ITT) analysis that analyses patients according to their allocations (i.e., regardless of the treatment received). This is meant to best approximate real-life situations, and outcomes are counted even if they are not measured. In contrast, in a per-protocol (PP) analysis, patients are assessed based on the treatment they received. ITT analyses are often more conservative and likely to detect a true treatment difference [26,27]. They are more similar to real-world results than PP. ITT analyses preserve the study design and statistical power as they are the same sample size as was initially designed. PP only includes patients if they follow protocol, and a PP analysis thus enhances the effect of treatment at the expense of real-world efficacy. A PP analysis reflects ideal circumstances and minimizes the impact of external causes. A PP analysis is often reported as a secondary analysis as even if the ITT analysis was negative overall, there is still interest in seeing if an effect exists.

### 3.2. Superiority versus Non-Inferiority Design

Non-inferiority studies aim to show that the effect of a new intervention is not worse than an active control. They are often used to assess two similar interventions in which one might offer an extra advantage, such as improved toxicity or lower cost. A non-inferiority study does not prove that two interventions are equal. A non-inferiority margin serves as a critical threshold, defining the degree of acceptable deterioration trialists will tolerate in their study. Deciding on a non-inferiority margin is a key statistical difficulty, and it is important to use clinical judgment in this decision. For non-inferiority studies, a one-sided 97.5% confidence interval must be calculated. The new treatment is deemed non-inferior if the confidence interval overlaps the non-inferiority margin. Given that the effect sizes of interest are much smaller in a non-inferiority study, typically, a larger sample size is needed than in a superiority trial [28].

A PP analysis is generally preferred for non-inferiority studies as it is more conservative since it is biased towards a difference [28]. More recently, there have been increased recommendations to transition towards ITT analyses for non-inferiority studies, with the aim of reducing missing variables.

## 4. Artificial Intelligence and Machine Learning in Clinical Trials Presented by Khaled El-Emam

**Figure 2:**
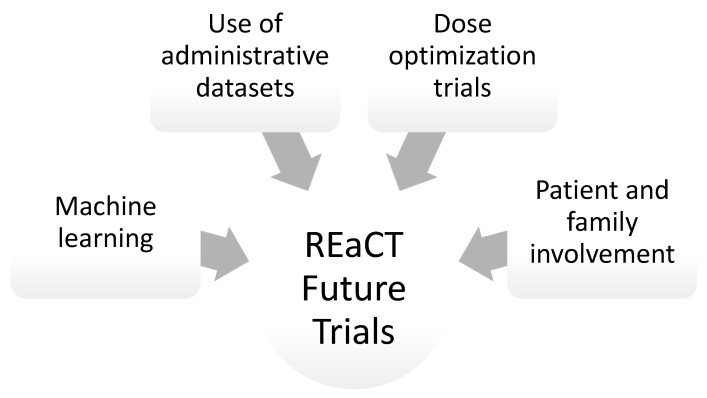
The future of REaCT.

The future of patient care and part of REaCT’s mandate centres on improved personalization of care. The emergence of machine learning can lead to significant advances towards the implementation of personalized medicine through the creation of algorithms to better tailor treatment and follow-up (Figure 2). Machine learning is artificial intelligence (AI) that allows computer systems to learn from experience without explicit programming and can use complex large datasets of multiple variables to learn the relationships between them and make predictions. Decision trees and artificial neural networks (ANN) are common machine learning algorithms. Artificial neural networks (ANNs) are models trained by processing examples to determine the relationships between various points in a dataset [29].

Machine learning algorithms have several strengths: they can model very complex functional forms and nonlinear relationships and handle missing data directly; they can discover complex interactions in the data; they can have multiple outputs simultaneously; and they can also deal with the high cardinality of categorical variables [30]. Some limitations of these algorithms are that they require large amounts of data, which are more than traditional statistical methods, and building models with small amounts of data can result in overfitting and model instability. There are no well-defined heuristics for sample size for machine learning. They are very sensitive to the quality of the data and possible measurement problems. Machine learning methods, specifically ANN outputs, are difficult to explain, representing a current challenge and work area [31].

Machine learning has been used to predict individual patient toxicities from cancer treatments. A study examining hot flashes in patients with breast cancer on endocrine treatment found that the perceived benefit of changes in hot flashes depends on the patient’s baseline value. For example, if a patient’s baseline hot flashes are severe, any reduction is perceived to be more impactful than in someone who has fewer hot flashes at baseline.

One potential use for machine learning is synthetic data generation. Eight randomized trials were recently used to train a generative model from a trained model to produce synthetic data, i.e., a new version of the datasets [32]. By employing sequential synthesis methods, researchers demonstrated that the synthetic data created could be a proxy for real clinical trial datasets.

Future avenues for machine learning include simulating additional control patients to increase the simulated patients’ diversity and addressing bias in clinical trials through simulating underrepresented groups. Machine learning can also be used to create algorithms and risk tools to help tailor follow-ups, improving care for patients. Future challenges to the use of machine learning will centre on the access to funding for academic institutions and the potential competing goals of private entities/industry.

## 5. Patient and Family Advisory Council Presented by Julie Renaud and Gwen Barton

The REaCT program engages patients and their loved ones by surveying them. This allows us to understand which clinical questions matter to them and the trial endpoints that they consider to be most critically important. To find novel ways to engage patients and their family and to foster better collaboration and partnership with patient advocates, we invited Julie Renaud and Gwen Barton to present on the Patient and Family Advisory Council (PFAC). The PFAC was founded as the Community Advisory Council in 2005 to provide advice, guidance and direction to the Regional Cancer Program.

The PFAC has a core group of 10 cancer patients or caregivers who meet monthly. The cancer program’s Person-Centred Cancer Care Manager and the Cancer Program Director support their meetings, activities and recommendations. An additional 20 patient and family representatives can be called upon if needed for their participation. A patient and family representative chairs the committee.

The involvement of the PFAC can be anywhere along the engagement continuum, which extends from communicating with and informing patients to partnering with them/co-design. It is important to foster engagement with patients and their families, and this can be established by involving patient and family advisors in planning, simplifying information, normalizing the sharing of information and considering equitable access to all.

## 6. The Use of Administrative Datasets in Clinical Trials Presented by Brian Hutton

Traditional approaches to patient follow-ups for clinical trials have limitations. For example, recurrence events are rare, and thus, large sample sizes are required, and routine monitoring for events translates to increased staffing time and study costs. These repeated follow-up visits also burden patients and can negatively impact their quality of life. Based on these factors, definitive trials are a challenge for the REaCT program with its mandate to gain efficiency and improve costs.

Routinely collected healthcare data (RCHD), also called “real-world data,” were presented as a potential future cost-effective approach to obtaining population follow-up data without the excessive demands of clinical follow-ups, all while improving patients’ quality of life. RCHD are data collected for reasons other than research or without specific research questions. The linkage of patient information with RCHD using unique identifiers was supported by patients with cancer, and its feasibility within Canada using IC-ES data sources has been proven [33].

Unlike hospitalization or death, RCHD do not formally capture disease recurrence, and creating a case definition for recurrence is necessary. REaCT was awarded a Canadian Cancer Society grant to conduct a scoping review to map and characterize case definitions for disease recurrence in breast cancer to help inform measures of this outcome in future trials [34]. The goal of this study includes identifying a collection of case definitions and assessments of their performance. We will also assess the feasibility of implementing RCHD within the REaCT setting and working to enhance the case definition to improve future performances. The REaCT group’s future project will use administrative datasets such as ICES data to re-capture patient outcomes for an ongoing pragmatic trial (REaCT-ZOL) and compare its findings with those captured using traditional REaCT follow-ups [35].

In the design of future trials, the REaCT program hopes to use administrative datasets to improve the follow-up process of clinical trials.

## 7. The Dose Optimization Trials and Future Perspectives for the REaCT Program Presented by Ian Tannock

The Optimal Cancer Care Alliance (OCCA) aims to influence regulators and guideline committees and advocate for the incorporation of requirements that compel companies to optimize the dosing and scheduling of new therapies. Additionally, the OCCA seeks to promote the initiation of dose optimization trials for drugs already in use. Many drugs are approved at doses that exceed those required to maximally inhibit their targets. Traditional trials are conducted to determine the maximally tolerated dose based on toxicity, and this high dose is carried over into later studies. Whether dose reductions or lower dosing intervals could lead to equivalent outcomes is unknown, and several cancer therapies can potentially lower costs and reduce toxicity if administered at a lower dose or with less frequent administration. This approach could enhance global access for all [36].

There are limitations to conducting non-inferiority optimization studies. As we discussed previously, one of these challenges is the larger sample size required for a non-inferiority study compared to that of a superiority study. A prevailing sentiment is that merely demonstrating non-inferiority in outcomes does not suffice. There should be a consideration of potential health outcomes’ superiority, such as reduced toxicity, improved quality of life or decreased costs. Furthermore, multiple outcomes should be encompassed in such a trial [37]. Near equivalence studies represent an alternative as they essentially widen the non-inferiority margin. They propose using all evidence, including clinical, pharmacokinetic and pharmacodynamic endpoints. There is often difficulty in obtaining funding for dose-finding studies despite these studies saving more money than they cost [38]. There is also difficulty in enrolment as patients and physicians fear the potential impact on treatment benefits [39,40]. Despite positive dose optimization trials, there is a low rate of implementation of low-dose strategies [41,42].

## 8. Discussion

The 2023 REaCT Retreat allowed clinicians and scientists to review past experiences and future avenues of the REaCT Program. An update on seven recently completed or currently accruing REaCT studies was presented. Clinical studies that aim to optimize widely used treatment delivery methods (REaCT-CHRONO and REaCT-RETT) generated great interest among patients and clinicians and yielded a rapid accrual. By using the integrative consent model, the patients enrolled in studies that compare standard-of-care treatments (REaCT-70, REaCT-5G, REaCT-HER TIME and REaCT-HOLD) have had a beneficial and empowering experience. However, the accrual of de-escalation studies (REaCT-70 and REaCT-HER TIME) is much slower possibly due to a more negative perception by physicians and patients. Also, these studies usually take more time to obtain consent from patients. Finally, most of the studies presented are multi-centre (REaCT-algorithm, REaCT-RETT, REaCT-CHRONO, REaCT-5G, REaCT-70 and REaCT-HOLD). It was observed that the peripheral sites accrued more slowly, which should be considered when evaluating the feasibility of a trial. A *REaCT Study Advisory Guideline* was created based on experience from current and past trials and will help evaluate the success of potential future studies (Supplementary Materials Table S1).

To better define the future direction of our program and improve its impact on global cancer care, some key statistical concepts, ways of analysing/collecting data and engaging patients were reviewed by a multidisciplinary panel of speakers. From the discussion generated by these presentations, multiple key areas of improvement were identified for the REaCT Program.

First, it is paramount that the REaCT Program finds ways to better engage with local and national organisations, policymakers and patient advocacy organizations to ensure that study findings are integrated in clinical practice guidelines. Furthermore, in the context of a publicly funded healthcare system, the next step would be to advocate for public institutions to provide funding for optimization trials which would help reduce unnecessary toxicity for patients as well as the cost of cancer care.

Additionally, the REaCT program must collaborate with other international academic institutions to open clinical trials in a collaborative setting. This will enhance visibility and the international impact of the REaCT Program. Also, this would help complete accrual in a timelier manner for pragmatic clinical trials that require a large number of patients. To avoid the administrative burden of regulatory requirements posed by different countries, one potential model of collaboration would be to have the same trial open and independently managed in other countries. Once completed, the data would then be combined.

Finally, the REaCT program should continue to leverage innovative trial design (near equivalence studies), machine learning and administrative datasets to improve the efficacy of trials.

## 9. Conclusions

In recent decades, clinical trials have shifted to predominantly pharmaceutical-company-funded investigational trials, generating multiple standards of care that have not been compared to each other. There is a need for inclusive, easy-to-conduct clinical trials to answer questions that are significant to both patients and healthcare providers. The REaCT program has a key framework to identify clinically relevant questions and simplify clinical trial protocols to replicate real-world practice as much as possible. The REaCT annual meeting reviewed current practice-changing REaCT trials, novel pragmatic trial methods and the improvement of engagement with cancer advocacy groups and other organizations. Future avenues for research projects were explored, which include the use of administrative datasets, machine learning and international collaboration.

## Figures and Tables

**Table 1:** Summary of key REaCT Studies.

Study	REaCT- Algorithm	REaCT-RETT	REaCT-CHRONO	REaCT-HER TIME	REaCT-5G	REaCT-70	REaCT-HOLD BMA	REaCT- Wellness
**Objective**	Assess how clinicians use PREDICT 2.1 to guide their decision making when ordering Oncotype Dx	Assess whether concomitant administration of ET throughout RT increases risk of toxicities	Assess whether the time of day of ET administration affects side effects and compliance	Assess feasibility of conducting larger trial examining 6 months of HER2-targeted therapy	Assess difference in bone pain between PEG and FIL in patients with early breast cancer receiving neo/adjuvant chemotherapy	Assess whether the omission of adjuvant ET for patients ≥ 70 with a lower-risk HR+ breast cancer (treated with standard loco-regional therapy) affects outcomes	Evaluate the optimal frequency of BMA use after 2 years of prior BMA treatment for bone metastases in patients with metastatic breast cancer and CRPC	Assess quality of life with de-escalated follow-up for patients with a history of breast cancer
**Arms**	N/A (before and after an educational intervention with PREDICT 2.1 results)	Sequential RT and ET vs. concurrent RT and ET	Morning ET dosing vs. evening ET dosing	N/A (single-arm study)	Five days of FIL vs. 1 dose of PEG	Omission of ET vs. administration of ET	Continuation of BMA every 4 or 12 weeks vs. de-escalating BMA to every 26 weeks	Guideline-based survivorship vs. annual phone call post-mammogram with on-demand access
**Enrollment**	Mar 2020– Nov 2021	Sep 17 2019–Jan 15 2021	Jun 2021– Mar 2022	Dec 2021–Present	Jun 2021–Mar 2023	Sep 2021–Present	Oct 2020–Present	Sep 2022– Mar 2023
**Number of patients enrolled**	620	262	245	20	233	72	171	244
**Endpoints**	Primary: proportion of patients for whom Oncotype Dx was ordered, defined as the number of patients with Oncotype DX orders divided by the number of patients eligible for Oncotype DX testing	Primary: ET toxicity per FACT-ES Secondary: FACT-TOI, EQ-5D-5L, RT toxicity and compliance	Primary: FACT-ES score from baseline to 12 weeks following the beginning of ET. Secondary: total score and individual items of FACT-ES and FACT-B from baseline to 4, 8, 12 and 52 weeks. Rates of discontinuation, interruption and compliance.	Primary: feasibility Secondary: cardiac events, Rx discontinuation EQ-5D-5L	Primary: bone pain during cycle 1 of chemotherapy Secondary: febrile neutro-penia rate, treat-ment-related hospitalizations, compliance, healthcare utilization, HR-QoL, cost utility, patient preference pre- vs. post-chemo	Primary: feasibility Secondary: significant AEs, PROs, treatment discontinuation and reasons	Primary: quality of life and physical function (C30+ BM22) at 48 weeks Secondary: Quality of life other time points, BMA toxicity, cost utility and EQ-5D-5L until year 1	Primary: quality of life by FACT-G 1 year post-randomization. Secondary outcome: fear of recurrence, anxiety levels, treatment-related toxicity, recurrences, frequency, type and reasoning behind any breast-cancer-related healthcare contacts, incremental cost-effectiveness ratio
**Results**	Results showed that the educational intervention did not impact molecular assay requests such as Oncotype DX. However, the study suggests that routine ordering of molecular assays for patients with low-clinical-risk disease is of poor value for most patients(9).	No difference in ET toxicity from baseline to 3 months and no difference in quality of life, ET compliance or RT toxicity at twelve months.	The 12-week FACT-ES scores mean changed from baseline and the proportion of patients with a clinically important decrease in their FACT-ES scores was not statistically different between the two groups. Secondary endpoint results, including 12-month adherence rate and quality of life were presented at a later symposium in 2023 (15).	N/A	No significant difference in patient-reported bone pain or quality of life between 5 days FIL and a single dose of PEG and no difference in chemotherapy delay, dose reduction or premature discontinuation. At the end of the study, preference for PEG increased in the PEG-treated group.	N/A	N/A	N/A
**Successes**	Important question potentially resulting in savings for the healthcare system, as well as substantial data generation regarding adjuvant decision making.	Strong multidisciplinary involvement, rapid recruitment of a large cohort and nimble response to pandemic restrictions (virtual recruitment and online questionnaire)	Incredible interest and engagement from patients and medical oncologists, as well as rapid accrual	Important question addressed, funding from CURE foundation, excellent patient identification and MD engagement	Developed in partnership with patient partners, using PROs as primary endpoint and strong engagement	First trial using integrated consent model to open in Saskatchewan. It had a steady rate of accrual, was designed for older patients and had good patient engagement	Trial caught attention of international advocacy group (GRASP) and had slow and steady accrual	Strong patient and physician engagement, as well as quick enrolment
**Challenges**	Impact of education intervention was minimal in changing clinical practice. Lower-than-expected accrual due to changes in practice due to COVID-19 pandemic.	Peripheral site activation and recruitment. Pandemic impacts on data collection and publication	Heavy workload for the CRA due to rapid accrual. Use of patient-reported outcomes as a primary endpoint	Small patient population, short window for enrollment, peripheral site activation and competing de-escalation studies	Peripheral site activation, completion of questionnaire pre-randomization, and heterogeneity across centres for chemotherapy regimens eligible for G-CSF prophylaxis	Time commitment to explain the study to patients, accrual in an older population, peripheral site recruitment and heavy workload due to being a mult-centre study	Peripheral site recruitment, recruitment of patients with CRPC due to short life expectancy and accuracy of data regarding toxicity for peripheral sites	Peripheral site recruitment and difficulty with data collection due to different follow-ups

## Data Availability

No new data were created. All study results will be published if available and data are available upon request.

## Supplementary Materials

The following supporting information can be downloaded at: https://www.mdpi.com/article/10.3390/curroncol31030104/s1, Table S1: REaCT Study Advisory Guideline.
