# Supplementary material for: The REthinking Clinical Trials Program Retreat 2023: Creating Partnerships to Optimize Quality Cancer Care
Source: Curr Oncol. 2024 Mar 6;31(3):1376–88. doi: 10.3390/curroncol31030104 (PMC10969202; doi:10.3390/curroncol31030104)
Supplement: Supplementary file 1 [file curroncol-31-00104-s001.zip › curroncol-2886124-supplementary.pdf]

Supplementary Materials

Table S1: REaCT Study Advisory Guidelines

**Collaboration & implication in the REaCT program by the PIs**	**√**
Actively participate in other REaCT trials	
Assist to quarterly study oversight meetings	
Provide regular update at the REaCT Program team meeting (attend 1x/ month and written communication weekly)	
**Pragmatic Design**	**√**
Integrative consent model	
Clinical equipoise of 2 standard interventions	
No additional visits than standard of care	
Inclusive eligibility criteria (limited number to reflect real-world-population)	
**Data collection**	**√**
Patient portal collecting PROs	
Limited additional data collection outside standard practice (limited adverse events, special events of interest, concurrent meds of interest)	
Reasonable follow up window	
Electronic database	
**Resources**	**√**
No additional tests (blood test, imaging, pathology)	
No impact or coordination with other departments/services (CTU, radiology)	
Limited number of participating sites that can commit to target accrual	
Study timing and workload is feasible with REaCT Program	
**Accrual**	**√**
Reasonable accrual rate (target 2 years) with interim accrual rate target at 1 year to proceed	
Multi-site study TOH should be able to accrue at least 60% of the sample size	
**Funding**	**√**
Funding obtained by PI (eg. grant, donation)	
Budget approved by REaCT Program prior to funding application	
**Trial Impact**	**√**
Improve patient care and/or resources (PROs, economic impact)	
Change clinical practice	
Must include a feasible KT plan (conference, manuscript, social media)	
